# Study on Metabolic Trajectory of Liver Aging and the Effect of Fufang Zhenzhu Tiaozhi on Aging Mice

**DOI:** 10.3389/fphar.2019.00926

**Published:** 2019-08-28

**Authors:** Duosheng Luo, Jingbiao Li, Kechun Chen, Yifan Yin, Zhaoyan Fang, Huiting Pang, Xianglu Rong, Jiao Guo

**Affiliations:** ^1^Key Unit of Modulating Liver to Treat Hyperlipemia SATCM (State Administration of Traditional Chinese Medicine), Guangdong Pharmaceutical University, Guangzhou, China; ^2^Guangdong Metabolic Disease Research Center of Integrated Chinese and Western Medicine, Guangzhou, China

**Keywords:** Fufang Zhenzhu Tiaozhi, metabolomics, liver aging, ultra-performance liquid chromatography, mass spectrometry

## Abstract

The aim of this study was to investigate the metabolic trajectory of liver aging, the effect of FTZ against liver aging in aging mice, and its mechanism using ultraperformance liquid chromatography/quadrupole-time-of-flight mass spectrometry (UPLC-Q-TOF/MS).

**Methods:** A total of 80 C57BL/6J Narl mice were randomly divided into five groups: 3-month-old group, 9-month-old group, 14-month-old group, 20-month-old group, and FTZ treatment group (20 months old). The mice in the treatment group received a therapeutic dose of oral FTZ extract (1.0 g/kg, on raw material weight basis) once daily during the experiment. The other groups received the corresponding volume of oral normal saline solution. Liver samples of all five groups were collected after 12 weeks, and UPLC-Q-TOF/MS was used to analyze metabolic changes. Orthogonal partial least squares–discriminant analysis (OPLS-DA) was used to analyze the resulting data. Additionally, cholesterol (TC), triglyceride (TG), aspartate aminotransferase (AST), alanine aminotransferase (ALT), secretion levels of TNF-α, IL-6, 5-LOX, and COX-2, as well as their relative mRNA expression in the liver were determined.

**Results:** The levels of TC, TG, AST, and ALT were increased, and liver tissue structure was damaged. The secretion levels of TNF-α, IL-6, 5-LOX, and COX-2, as well as their relative mRNA expression in the liver also increased with aging. FTZ administration reduced the symptoms of liver aging. The OPLS-DA score plot illustrated the effect of FTZ against liver aging, with N-acetyl-leukotriene E4, 20-hydroxy-leukotriene E4, leukotriene E4, and arachidonic acid among the key biomarkers. The pivotal pathways revealed by pathway analysis included arachidonic acid metabolism and biosynthesis of unsaturated fatty acids. The mechanism by which FTZ reduces the symptoms of liver aging in mice might be related to disorders of the abovementioned pathways.

**Conclusion:** A metabolomic approach based on UPLC-Q-TOF/MS and multivariate statistical analysis was successfully applied to investigate the metabolic trajectory of liver aging. FTZ has a protective effect against liver aging, which may be mediated *via* interference with the metabolism of arachidonic acid, biosynthesis of unsaturated fatty acids, and downregulation of pro-inflammatory factors in the liver in mice *in vivo*.

## Introduction

As the global population continues to age, the health status of middle-aged and elderly people continues to decline, which is likely to increase age-related health burdens such as hypertension, diabetes, and cardiovascular and other chronic diseases (De Luca d’Alessandro et al., 2011). According to a recent study of the glucose metabolism status in middle-aged and elderly people, the pre-diabetes and diabetes prevalence rates were increasing with age (Song, 2017). Similarly, the TC, TG, and LDL-C levels were found to increase, while the HDL-C levels showed a downward trend with age (Lawton and Berger, 2008; Gregg et al., 2012). The liver is an important metabolic center for the regulation of glucose and lipid homeostasis, while also being a detoxification site playing an important role in maintaining the homeostasis and improving the function of the whole body (Grizzi et al., 2013). Studies have also shown that liver aging is associated with liver regeneration, stress response, and inflammatory response (Gan et al., 2011). Liver aging is closely related to the severity and progression of nonalcoholic fatty liver disease, nonalcoholic steatohepatitis, and metabolic syndrome (especially diabetes and obesity) (Frith et al., 2008; Honma et al., 2011; Tomás-Loba et al., 2012; Wuttke et al., 2012).

Metabolomics analyzes changes in the abundance of the totality of small molecules to establish a relative relationship between endogenous substances and disease physiology. Blood, urine, and tissue extracts were used to confirm the endogenous changes under certain *in vivo* conditions, and the mechanism by which the investigated drugs exerts their influence (Plumb et al., 2005). Therefore, it is of great significance to apply metabolomics to the study of the aging processes and the related drug research. However, there are few reports on using metabolomics to characterize the metabolic trajectories of the liver during aging.

The liver is an important metabolic center for the regulation of glucose and lipid homeostasis. FuFang Zhenzhu TiaoZhi (FTZ) is an effective traditional Chinese medicine which has been prescribed for 20 years. It based on the theory of “modulating liver, starting pivot, and cleaning turbidity” and has proven clinical efficacy as a prescription for patients with dyslipidemia, and those requiring glucocorticoid, but its effect on liver aging is unclear (Guo et al., 2011; Hu et al., 2014). This study is the first to our knowledge to investigate the changes of the liver metabolome during the normal aging process in mice, as well as the effect of FTZ against liver aging.

## Materials and Methods

### Preparation of the FTZ Extract

Herbs in FTZ (Citri sarcodactylis fructus, Ligustri lucidi fructus, Salviae miltiorrhizae radix et rhizoma, Notoginseng radix et rhizoma, Coptidis rhizoma, Atractylodis macrocephalae rhizoma, Cirsii japonici herba et radix and Eucommiae cortex) were provided by Zhixin Chinese Herbal Medicine Co., Ltd. (Guangzhou, China) and authenticated by Professor Wei He, Guangdong Pharmaceutical University. FTZ was obtained from the Institute of Materia Medica, Guangdong Pharmaceutical University. The voucher specimen was GDPUZYY 20110901-8 (**Supplementary Material-Voucher** specimen) (Guo et al., 2011). Quality analysis of the FTZ extract was performed via HPLC fingerprinting (Zhong et al., 2012).

### Animals and Experimental Design

100 specific pathogen-free (SPF) C57BL/6J Narl mice (4 weeks, body weight 18–23 g) purchased from the same vendor were obtained from Guangdong animal experimental medical center. The mice were housed in a temperature-controlled room at 25 ± 2°C. During the experiment, standard solid food and water were provided. The study was reviewed and approved by the Animal Ethical Committee of Guangdong pharmaceutical University (No. gdpulac20140176). After acclimatization for 1 week, 100 mice were randomly divided into five groups (20 mice per group) in respective ages (3-, 9-, 14-, 20-month-old groups and 20-month-old treatment group). The treatment group was orally gavaged FTZ at a dose of 1.0 g kg^-1^ d^-1^ every day for 12 weeks continuously; the other group was orally gavaged the same volume of normal saline during the experimental period.

### Assay of Body Weight and Organ Indices

At 12 weeks after the start of treatment, the mice were fasted for 14 hours before dissection, and under the anesthesia of ether, blood was collected from the ocular venous plexus. Blood was centrifuged at 3,000 rpm for 10 min, after which the serum was collected and stored at −80°C. The liver, thymus, and spleen were collected, washed with phosphate buffered saline, weighed, and stored in a freezer tube at -80°C. The organs were weighed to calculate the organ coefficients. Parts of the fresh liver tissues were removed and kept frozen for biochemical measurements and histopathological assays.

### Determination of TC, TG, AST, and ALT in the Liver

Levels of TC and TG in the liver were measured using a TC and TG Kit (Rongsheng Biological Co., Ltd., China). The levels of AST and ALT in serum were assessed using ELISA Assay Kit (CUSABIO, Nanjing, China) in accordance with the manufacturer’s instructions.

### Liver Metabolomics

An aliquot comprising exactly 50 mg of liver tissue was freeze-dried for 12 h, suspended in 500 μl of 80%, homogenized at 5,000 rpm for 25 s, left to stand for 10 min, and then centrifuged at 15,000 rpm for 15 min at 4°C. The clear supernatant was moved to a new EP tube for UPLC-Q-TOF/MS analysis. All samples were analyzed in both positive and negative ion modes.

An ACQUITY UPLC BEH C18 column (100 mm ×2.1 mm, 1.7 μm; Waters, USA) and a guard column (Waters, USA) were used at 30°C column temperature. Injection volume was 5 µl in both positive and negative ion modes. The mobile phase consisted of 0.1% formic acid (Merck, Germany) in water as solvent A and acetonitrile (Merck, Germany) as solvent B in gradient elution mode (0–3 min, 98–75% A; 3–8 min, 75–50% A; 8–15 min, 50–30% A; 15–20 min, 30–12% A; 20–25min, 12–0% A). The flow rate of the mobile phase was 0.4 ml min^-1^.

The UPLC system was interfaced with a AB SCIEX TripleTOF 5600 mass spectrometer (AB Science, United States) with the following settings: positive ion mode: ion spray voltage, 5,500 V; ion source temperature, 500°C; decluttering potential, 80 eV; collision energy, 10 eV; collision energy spread, 20 eV; and the curtain gas, ion source gas 1 and gas 2 were set at 50 psi. Negative ion mode: ion spray voltage, 4,500 V; ion source temperature, 500°C; decluttering potential, -100 eV; collision energy, -10 eV; collision energy spread, 20 eV; and the curtain gas, ion source gas 1 and gas 2 were set at 50 psi. The information dependent acquisition (IDA) mode (Chen et al., 2009) was used to qualify the potential biomarkers in both positive and negative ion modes.

### Analysis of TNF-α, IL-6, 5-LOX, and COX-2

The total liver RNA was extracted using RNAiso Plus Kit (TaKaRa, Japan), reverse transcribed into cDNA using TB Green^™^ *Premix Ex Taq* ^™^ II Kit (TaKaRa, Japan), and amplified using a Piko Real real-time quantitative PCR instrument (Thermo-Fisher Scientific, USA). The secretion of TNF-α, IL-6, 5-LOX, and COX-2 in the liver was detected using a mouse immunoassay kit (Wuhan Huamei Biotechnology Co., Ltd.).

### Statistical Analysis

All comparisons between two groups of measurements were made using Student’s *t*-test, and comparisons between multiple groups were made using ANOVA followed by Bonferroni’s multiple *t*-test (Shiota et al., 2013). Statistical significance was defined as being reached at *p* < 0.05. All data are expressed as the means ± standard deviation (SD). Statistical significance of the data was analyzed using Prism GraphPad 6 Software (GraphPad Software Inc., San Diego, CA, USA). The LC-MS raw data was analyzed using MarkerView software (USA, AB SCIEX) and then was introduced to SIMCA-P software (version 13.0, Umetrics, Umeå, Sweden) for orthogonal partial least square–discriminant analysis (OPLS-DA). The goodness-of-fit parameter (R^2^X) and the predictive ability parameter (Q^2^) were used to analyze the quality of the models. At the same time, the differential markers were matched and identified using PeakerView software (USA, AB SCIEX).

## Results

### Food Intake, Body Weight, and the Wet Weight Coefficient of Organs

**Figure 1:**
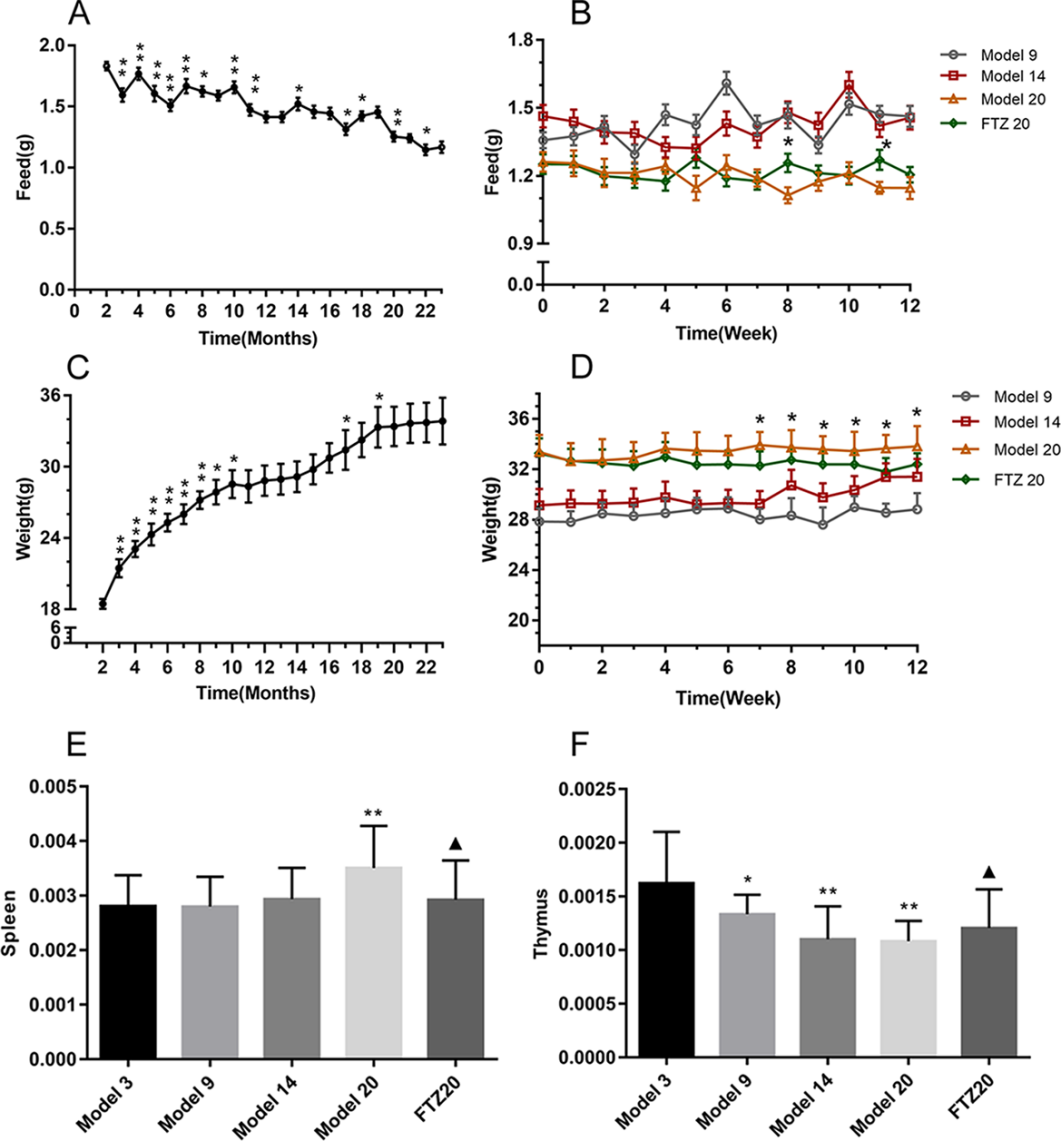
**(A–B)** Feed intake, **(C–D)** body weight, and **(E–F)** tissue wet weight coefficient of mice. All data were expressed as the means ± SD. *P < 0.05, **P < 0.01 compared to model 3 group; ^▲^P < 0.05 compared to model 20 group.

As the age increased, the food intake decreased, as shown in **Figure 1A**. During the FTZ treatment period, there was no significant difference in the food intake between the FTZ group and the 20 model groups, as shown in **Figure 1B**. The body weight, which showed an increase with age, was not affected by the amount of food (**Figure 1C**). Compared with the 20 model groups, the body weight of the FTZ group decreased significantly after 7 weeks of treatment (**Figure 1D**), indicating that FTZ has the effect of reducing weight.

The spleen and thymus are important immune organs, and the wet weight coefficient of spleen increased with age. However, in the FTZ group, it was significantly lower than in the same age group without treatment. The wet weight coefficient of the thymus decreased with age, while in the FTZ group, it increased significantly, as shown in **Figures 1E, F**.

### Levels of TG, TC, AST, and ALT in the Liver

**Figure 2:**
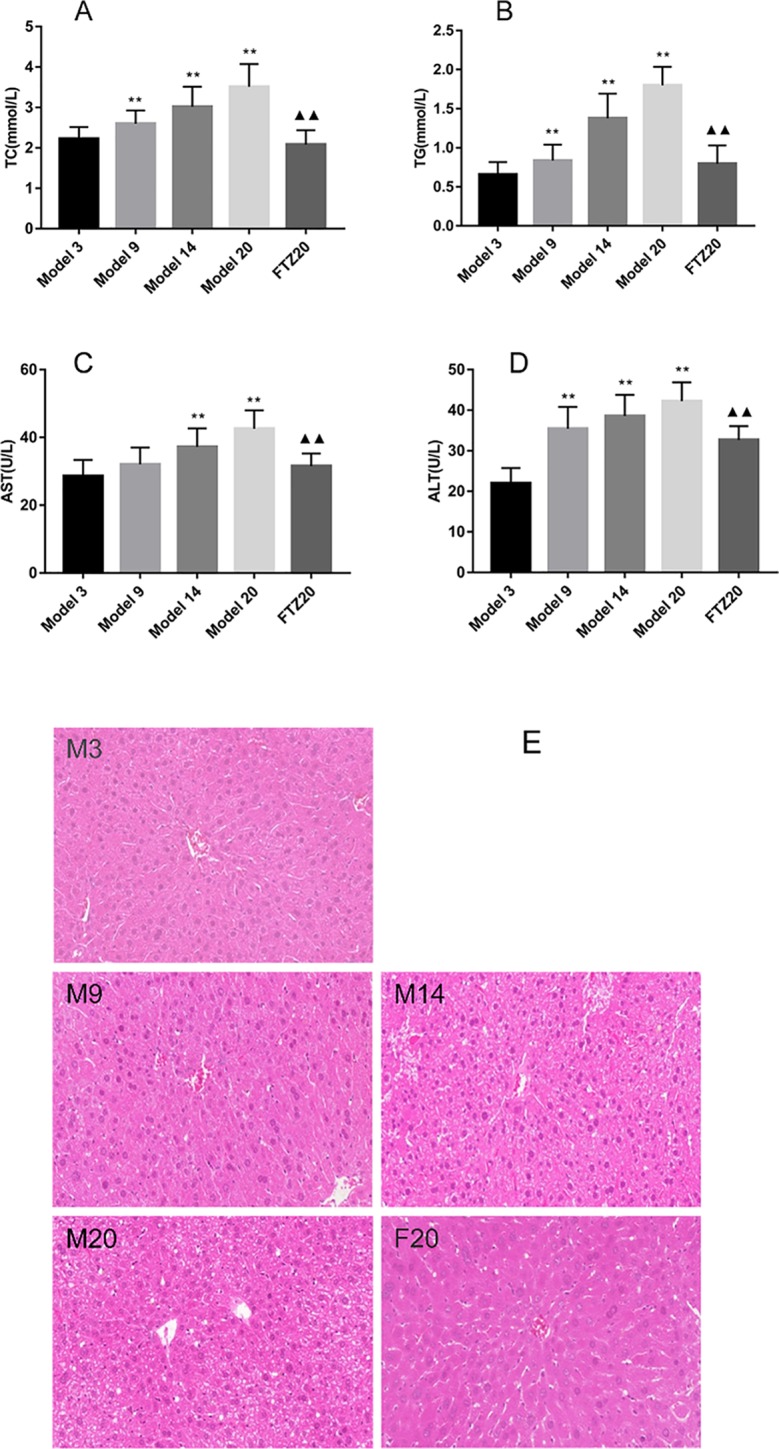
**(A–B)** TG and TC contents in liver of aging mice. **(C–D)** Liver function indicators AST and ALT levels. **(E)** Mouse liver pathology H&E staining (100x). **P < 0.01 compared to model 3 group; ^▲▲^P < 0.01 compared to model 20 group.

The TG and TC contents in the liver increased with age. However, in the FTZ group, it decreased significantly compared with the untreated 20 model groups (**Figures 2A, B**). The contents of AST and ALT in the serum increased with age, while it decreased significantly with FTZ treatment, as shown in **Figures 2C, D**.

With the increase of age, liver damage increased significantly. The liver structure at 20 months old was more pronounced, and the central vein was blurred; the normal liver cells were surrounded by loose swelling, and there was infiltration of inflammatory cells. With FTZ treatment, the hepatic lobule was structurally intact, the hepatic cords were arranged neatly, the decomposition was clear, and the hepatocytes were slightly swollen, while no obvious infiltration of inflammatory cells was observed (**Figure 2E**), indicating that FTZ may improve liver structure and function.

### Liver Tissue Metabolomics

**Figure 3:**
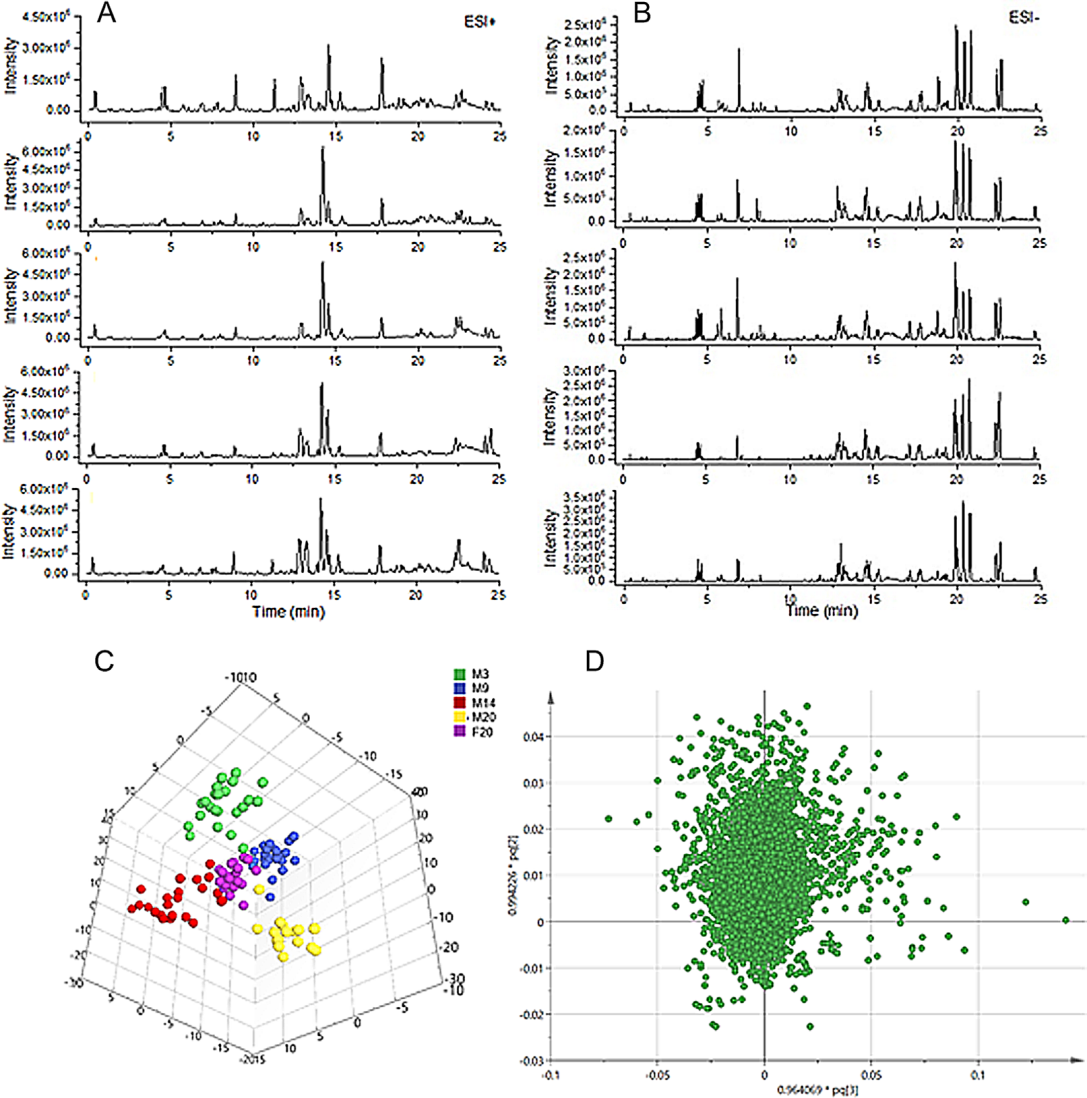
Profiling and multivariate statistical analysis. **(A)** Base-peak intensity chromatograms of different groups in positive ion mode obtained from UPLC-TOF/MS analysis. **(B)** Base-peak intensity chromatograms of different groups in negative ion mode obtained from UPLC-TOF/MS analysis. **(C)** Score plot of OPLS-DA of different groups (R^2^Y = 0.869, Q^2 =^ 0.779). **(D)** Loading plot of OPLS-DA in positive ion mode from different groups.

The data demonstrated good reproducibility of the UPLC-MS/MS method used in this study. Typical base-peak intensity chromatograms are shown in **Figures 3A, B**. Score plots of the OPLS-DA model are shown in **Figure 3C** (ESI^+^ R^2^Y = 0.782, Q^2^ = 0.505: ESI^-^ R^2^Y = 0.869, Q^2^ = 0.779). The score maps effectively distinguished between different age groups and the FTZ group.

To identify the significantly altered metabolites, their contribution to the clustering was assessed according the variable importance values of more than 1.5 in the loading plot of the OPLS-DA model (**Figure 3D**) and *p*-values of less than 0.05. The identified potential biomarkers were listed in **Table 1**. Structures corresponding to the selected metabolites were obtained by searching the freely accessible HMDB (http://www.hmdb.ca) and KEGG (http://www.genome.jp) databases. Finally, 25 differentially abundant metabolites were identified, encompassing seven related to fatty acid metabolism, six related to bile acid metabolism, five related to arachidonic acid metabolism, four related to phospholipid metabolism, and two markers related to amino acid metabolism.

**Table 1:** Identified potential biomarkers in liver and fold changes (FC) among different groups.

No.	Maker	VIP	FC (M9/M3)	FC (M14/M3)	FC (M20/M3)	FC (F20/M20)
1	Taurocholic acid	11.02	0.90	1.44	2.49	0.53
2	Bovinic acid	9.08	1.76	1.03	1.28	1.19
3	15-Hydroxyicosanoic acid	8.74	1.72	1.31	1.39	1.35
4	Arachidonic acid	7.95	1.59	1.17	1.08	1.63
5	Palmitoleic acid	5.20	2.07	1.16	1.34	1.06
6	γ-Linolenic acid	4.34	2.14	1.18	1.73	0.84
7	LysoPE (16:0/0:0)	3.86	1.17	1.11	1.36	0.78
8	20-HETE	3.51	2.86	1.56	3.08	0.10
9	3b,7a,12a-Trihydroxy-5b-cholanoic acid	3.50	1.27	3.56	7.05	0.57
10	LysoPE (0:0/24:0)	3.40	1.06	0.90	0.82	1.14
11	PA (P-16:0e/18:2(9Z,12Z))	3.38	2.05	1.36	1.65	1.33
12	LysoPC (16:0)	3.25	0.99	1.05	1.01	1.11
13	Glycerol triundecanoate	2.83	2.39	1.74	3.07	0.70
14	7-Ketodeoxycholic acid	2.76	0.30	10.26	15.70	0.93
15	N-Acetyl-leukotriene E4	2.71	1.11	1.04	1.19	1.09
16	3-Oxocholic acid	2.65	0.90	3.27	4.77	0.31
17	12-HETE	2.45	1.05	0.91	1.29	1.14
18	20-Hydroxy-leukotriene E4	2.30	1.18	1.95	3.79	0.55
19	Docosahexaenoic acid	2.16	1.25	1.50	0.47	5.42
20	6-Phosphogluconic acid	2.04	1.14	1.24	0.87	1.97
21	17-Hydroxylinolenic acid	1.99	1.20	0.76	1.18	1.48
22	11,14,17-Eicosatrienoic acid	1.96	1.60	1.23	1.12	1.74
23	D-Glutamine	1.87	0.26	0.73	0.39	2.91
24	γ-Glutamylcysteine	1.87	2.59	1.06	2.09	0.63
25	Taurodeoxycholic acid	1.76	0.28	0.63	0.78	0.62

When the variance was homogeneous, the LSD method was used for multiple analysis. When the variance was not uniform, the Games-Howell method was used for multiple analysis.

VIP values were obtained from the model.

Fold change (FC) was calculated based on a binary logarithm for model vs. control and FTZ vs. model. FC with a value greater than zero indicates a higher intensity of the plasma metabolite, while a FC value less than zero indicates a lower intensity of the plasma metabolite.

**Figure 4:**
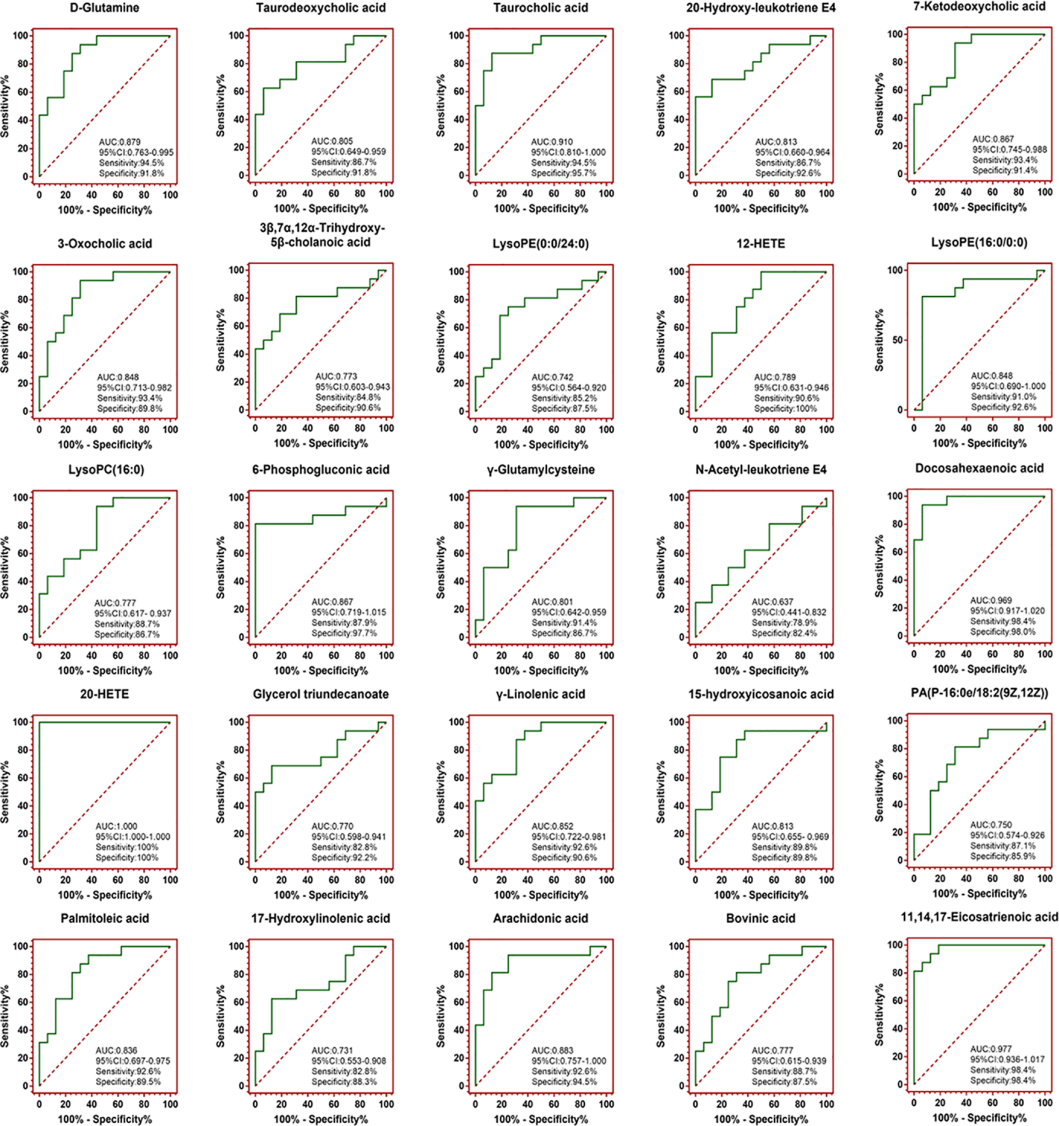
OPLS-DA-based ROC curves of the 25 potential biomarker of anti-liver aging effects of FTZ. The associated AUC, 95% CI, sensitivities, and specificities were indicated.

**Figure 5:**
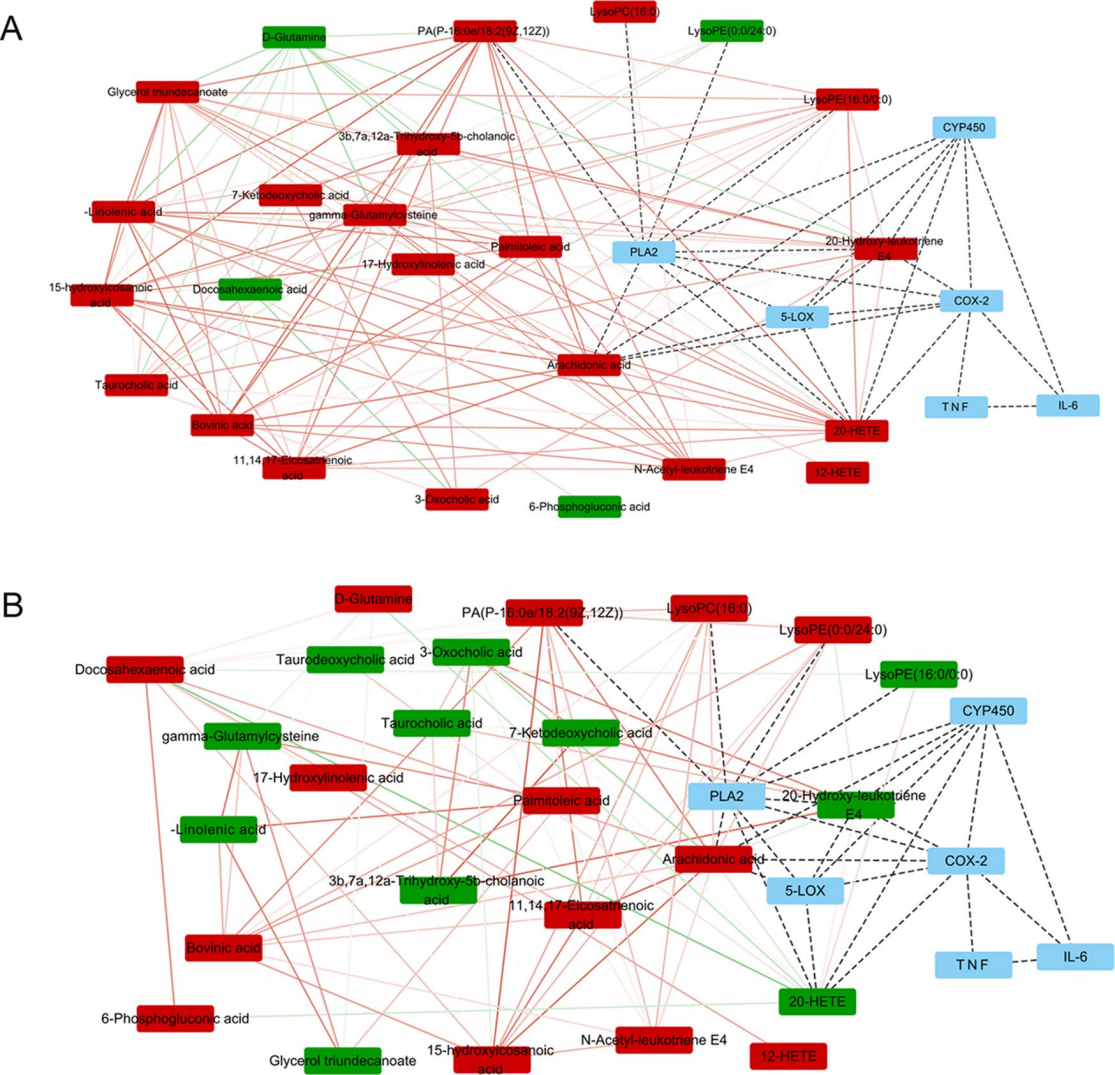
Metabolite pathway analysis with Pearson correlation coefficient only edges corresponding to correlations whose similarity was less than 0.5 are shown. **(A)** Metabolite pathway analysis between M3 and M20. **(B)** Metabolite pathway analysis between M20 and F20. The upward adjustment is red; the downward adjustment is green. The edges red solid line represents positive correlation, and the green solid line represents negative correlation. The line between metabolism–protein and protein–protein is indicated by a black dotted line.

Potential biomarkers of the effect of FTZ against liver aging were searched using OPLS-DA-based ROC curves. The 25 biomarkers shown in **Figure 4** had high sensitivity (> 90%), specificity (> 90%), and AUC values (> 0.80). The metabolic pathways and networks possibly influenced by aging were searched *via* MetPA analysis (**Table 2** and **Figure 5**).

**Table 2:** Ingenuity pathway analysis with MetPA from differential biomarkers.

Pathway name	Total metabolites	Hits	p	-Log(*p*)	Holm *p*	FDR	Impact	Details
Biosynthesis of unsaturated fatty acids	42	3	0.0104	4.57	0.85	1.00	0.000	KEGG
D-Glutamine and D-glutamate metabolism	5	1	0.0553	2.89	1.00	1.00	0.000	KEGG
Arachidonic acid metabolism	36	2	0.0602	2.81	1.00	1.00	0.326	KEGG
Taurine and hypotaurine metabolism	8	1	0.0870	2.44	1.00	1.00	0.000	KEGG
Ether lipid metabolism	13	1	0.1378	1.98	1.00	1.00	0.000	KEGG
Pentose phosphate pathway	19	1	0.1952	1.63	1.00	1.00	0.047	KEGG
Glutathione metabolism	26	1	0.2576	1.36	1.00	1.00	0.078	KEGG
Glycerophospholipid metabolism	30	1	0.2912	1.23	1.00	1.00	0.044	KEGG
Primary bile acid biosynthesis	46	1	0.4119	0.89	1.00	1.00	0.030	KEGG

“Total” is the total number of differential metabolites species in the pathway; “hits” is the actually matched number from the user differential biomarkers; the raw p is the original p calculated from the enrichment analysis; the “Holm p” is the p value adjusted by Holm−Bonferroni method; “impact” is the pathway impact value calculated from pathway topology analysis.

### Secretion Levels of TNF-α, IL-6, 5-LOX, and COX-2 and Their Relative mRNA Expression in the Liver

**Figure 6:**
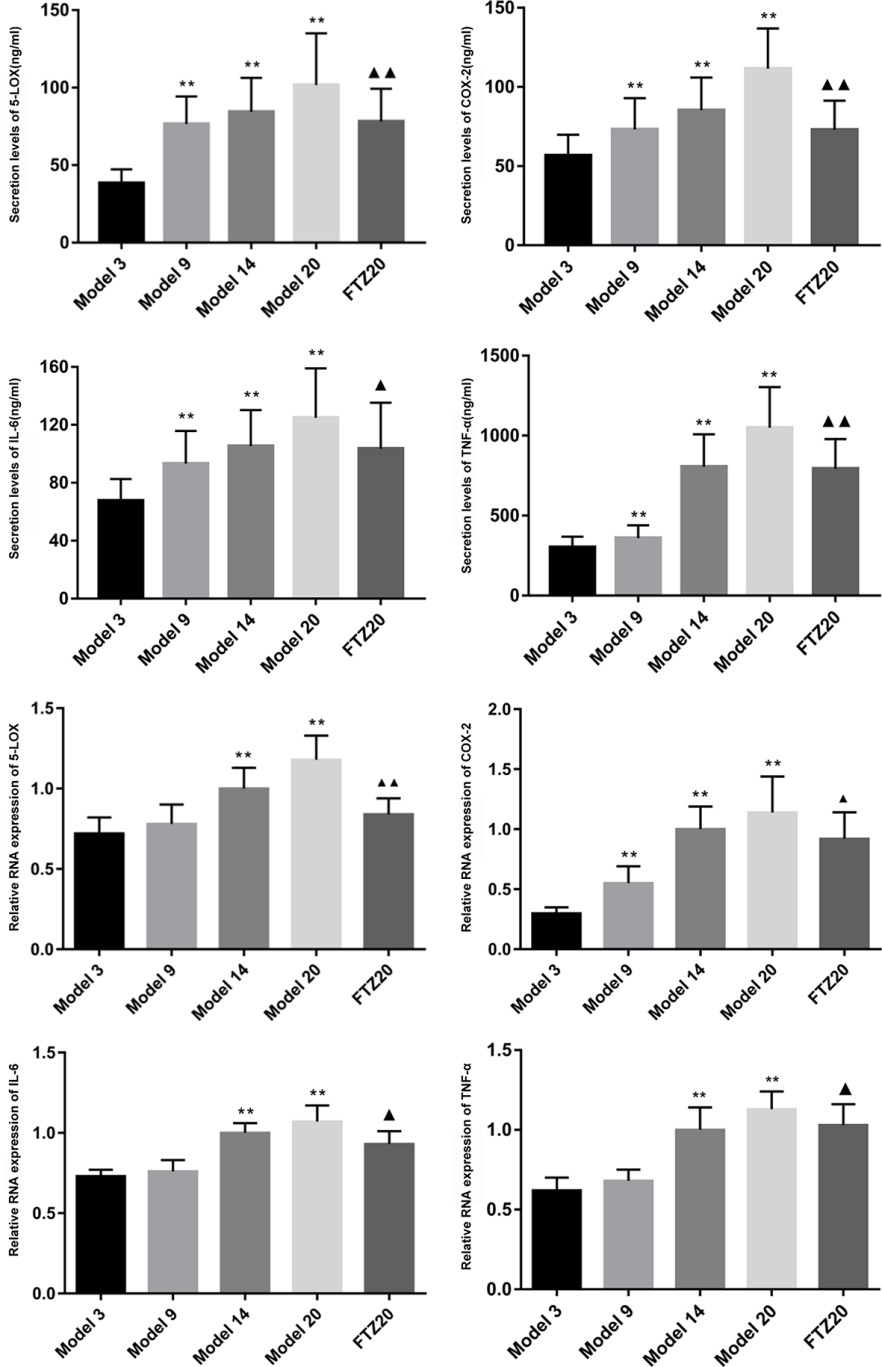
Secretion levels of TNF-α, IL-6, 5-LOX, and COX-2 and relative mRNA expression in liver.All data were expressed as the means ± SD. **P < 0.01 compared to model 3 group; ^▲^P < 0.05, ^▲▲^P < 0.01 compared to model 20 group.

With aging, the mRNA expression of TNF-α, IL-6, 5-LOX, and COX-2 in the liver increased significantly. With FTZ treatment, it decreased significantly compared to the 20 model groups. The results showed that these changes were accompanied by inflammation during the aging process, and that the inflammation was effectively controlled by the FTZ treatment. The levels of TNF-α and IL-6 increased significantly with age and decreased significantly with FTZ treatment. The secretion levels of 5-LOX and COX-2 increased significantly with the increase of age, while in the FTZ group, they were significantly decreased compared to the 20 model groups (**Figure 6**).

## Discussion

The liver plays a central role in metabolism, and studies have shown that insulin resistance associated with dysregulation of lipid metabolism in the elderly can lead to an increased stress response which significantly decreases this organ’s various functions, causing a variety of liver diseases. Therefore, studying the physiological function and metabolism of the liver is crucial for understanding the mechanism of aging and evaluating the efficacy of drugs (Rajendrasozhan et al., 2006).

Lipid metabolism and aging show a significant correlation in the liver, and abnormalities of lipid metabolism are generally more serious with age (Tomás-Loba et al., 2012). In this study, the lipid metabolism in the livers of aging mice changed significantly, and a total of seven differentially abundant lipid metabolites were identified. Conversely, the fatty acid metabolism improved significantly with FTZ treatment. The decrease of cholesterol may be related to the regulation of cholesterol ester acylase and cholesterol ester hydrolase activity (Quehenberger et al., 2010). Our own research revealed that FTZ can regulate cholesterol ester acylase (Cao et al., 2012). At the same time, the study showed that with the increase of age, the content of cholic acid increased significantly. The level of cholesterol increased with age, and bile acids are the end-product of cholesterol catabolism (Staley et al., 2016), indicating that bile acid metabolism and cholesterol metabolism disorders occurred with increasing age, and FTZ treatment partially reversed these bile acid abnormalities.

The arachidonic acid (AA) metabolites showed significant differences with age, indicating that AA metabolism plays an important role in liver aging. When pro-inflammatory factors enter cells, phospholipids are hydrolyzed by phospholipase A2 (PLA2), which triggers the inflammation cascade, which involves the metabolism of AA (C Meyer et al., 2005). AA metabolism forms the main core of inflammatory metabolism (Meyer et al., 2005), in which it is converted into inflammatory mediators such as leukotrienes and prostaglandins through cyclooxygenase (COX), lipoxygenase (LOX), and cytochrome P450s (CYP450s) (Salminen et al., 2008). Inflammatory mediators stimulate mast cells and macrophages to release pro-inflammatory factors such as IL-1β, TNF-α, etc., triggering the inflammatory response, and the body’s inhibition of inflammation is gradually weakened, eventually leading to the chronic inflammation associated with aging (Giunta and Sergio, 2009; Wang et al., 2016). The liver is not only an important site of the production of AA metabolites, but also the major organ of metabolic inactivation properties.

The metabolomics results showed that the content of the potential markers N-acetyl-leukotriene E4, 20-hydroxy-leukotriene E4, and leukotriene E4, which are lipid mediators that are generated from AA when it is metabolized by LOX, increased significantly with age. LTs are mainly metabolized, inactivated, and excreted by the liver and gallbladder. The 5-LOX pathway produces LTB4 which enhances the production of chemokines and causes leukocytes to accumulate in the inflammation site. With age, the liver metabolic function of LTs decreases, and LTs cause liver cell lesions, which may cause liver tissue damage and accelerate liver aging (Keppler et al., 1985). Cyclooxygenase-2 (COX-2) is one of the key enzymes required for AA metabolism in the COX pathway. COXs convert AA into prostaglandins (PG), which promote the inflammatory response. COX-2 is an inducible enzyme that is expressed only at low levels under normal conditions, but can be strongly induced by inflammatory mediators, and in turn produces pro-inflammatory effects (Samad et al., 2001). COX-2 can catalyze the synthesis of PGs from AA to participate in the inflammatory response, but the production and expression of COX-2 are regulated by pro-inflammatory factors such as TNF-α and IL-1β (Narita et al., 2008). In chronic inflammation, LTs and PGs have synergistic effects, so that drug interventions need to inhibit both COX and 5-LOX activities, thereby preventing the formation of both PGs and LTs (Yang et al., 2007).

The normal aging process is accompanied by chronic inflammation, while the liver is the metabolic center of the body, and its degenerative aging leads to an accumulation of defects in the glycolipid metabolism and increases the risk of disease (Keppler et al., 1985; Moore et al., 1990). Franceschi et al. reported that the inflammation increases with age, while the anti-inflammatory mechanisms are weakened, and first proposed inflammatory aging (Fraceschi et al., 2000). Salvioli et al. found that pro-inflammatory cytokines play an important role in inflammatory aging (Salvioli et al., 2013). Elevated levels of the pro-inflammatory factors IL-6 and TNF-α in human serum can be used as predictors of inflammatory aging in the elderly (Bonafè et al., 2011). Studies have found high levels of pro-inflammatory cytokines, causing inflammatory stress, leading to tissue aging, and aging-related diseases in the elderly (De martinis et al., 2005). Morphological and functional changes associated with liver aging include lipid accumulation, swelling, and increased levels of pro-inflammatory factors (Salminen et al., 2008).

In this study, the metabolomics results showed that the potential markers in the AA metabolic pathway were mainly leukotrienes, which increased significantly. Similarly, the levels of IL-6, TNF-α, and the relative expression of their mRNAs increased with age, indicating that liver aging and chronic inflammation occurred during the normal aging process in mice. With FTZ treatment, the levels of leukotrienes decreased, whereby the expression levels of TNF-α, IL-6, 5-LOX, and COX-2 were down-regulated, indicating that the AA metabolic pathway was inhibited by FTZ treatment.

In summary, we studied the metabolic trajectory of liver aging and the effect of FTZ on the livers of aging mice for the first time and showed that the effect of FTZ against liver aging may be related to the regulation of the biosynthesis of unsaturated fatty acids, AA metabolism, and downregulation of pro-inflammatory factors in the liver.

## Conclusions

A metabolomic approach based on UPLC-Q-TOF/MS and multivariate statistical analysis was successfully applied to investigate the metabolic trajectory of liver aging and the protective effects of FTZ against liver aging in mice. Furthermore, the TG and TC contents in the liver increased, liver damage increased, and secretion levels of TNF-α,IL-6, 5-LOX, COX-2, as well as their relative mRNA expression in the liver, also increased with age. FTZ has a protective effect against liver aging, which may be mediated *via* interference with the metabolism of AA, biosynthesis of unsaturated fatty acids, and downregulation of pro-inflammatory factors in the liver in mice *in vivo*. This study provides evidence that FTZ may serve as a potential therapeutic agent for the treatment of normal liver aging.

## Data Availability

All datasets generated for this study are included in the manuscript and the supplementary files.

## Ethics Statement

This study was carried out in accordance with the recommendations of regulations of the experimental animal ethics committee of Guangdong Pharmaceutical University. The protocol was approved by the experimental animal ethics committee of Guangdong Pharmaceutical University.

## Author Contributions

JG, XR and DL were responsible for the conception and design of the study. DL, JL and KC were responsible for the data collection, analysis, image processing and writing the manuscript. All authors read and approved the final manuscript.

## Funding

This study was supported by a grant from the National Natural Science Foundation of China (Grant No. 81503313, 2015) and a research project with the Guangdong Province Natural Science Fund (Grant No. 2016A030313739 and Grant No.2016B050501003).

## Conflict of Interest Statement

The authors declare that the research was conducted in the absence of any commercial or financial relationships that could be construed as a potential conflict of interest.
