# Supplementary material for: Study on Metabolic Trajectory of Liver Aging and the Effect of Fufang Zhenzhu Tiaozhi on Aging Mice
Source: Front Pharmacol. 2019 Aug 28;10:926. doi: 10.3389/fphar.2019.00926 (PMC6722462; doi:10.3389/fphar.2019.00926)
Supplement: Supplementary file 1 [file Presentation_1.pdf]

Supplementary Material

S1. Protocol for the preparation of FTZ extract.

The FTZ was prepared by alcohol and water extraction of 8 herbs as following described.

2. 4 kg herb material of FTZ-2 (composed of Ligustri Lucidi Fructus and Coptidis Rhizoma in a ratio of 3: 1) was refluxed with 70% ethanol twice (24 L, and 19.2 L) for 2 h respectively. The refluxes was filtered and then concentrated *in vacuo* at 60 °C to a concentration of 1.2 g/ml (w/v) to render the FTZ-2 extract.

7 kg herb material of FTZ-3 (composed of Atractylodis Macrocephalae Rhizoma, Salviae Miltiorrhizae Radix et Rhizoma, Citri Sarcodactylis Fructus, Eucommia Cortex, Rhizoma Cirsii Japonici in a ratio of 6:5:5:4:3) was decocted in 84 L water for 1.5 h. The suspension was then filtered and 63 L water was added for the second decoction lasted 1 h. The filtered and mixed suspension from two decoctions was then concentrated *in vacuo* at 60 °C to a concentration of 1.2 g/ml (w/v) to render the water extract.

0. 6 kg Notoginseng Radix et Rhizoma was refluxed with 50% ethanol twice (6 L and 4.8 L respectively) for 2 h. The refluxes was filtered and then concentrated *in vacuo* at 60 °C to a concentration of 1.2 g/ml (w/v) to render the Notoginseng extract.

The above three liquid extracts were mixed and then concentrated to dryness *in vacuo* at 60 °C to render the final FTZ extract at an yield of 25% (w/w).
